# Sleep Disorders and Atrial Fibrillation: Current Situation and Future Directions

**DOI:** 10.22086/gmj.v0i0.1416

**Published:** 2018-11-24

**Authors:** Alireza Sepehri Shamloo, Arash Arya, Nikolaos Dagres, Gerhard Hindricks

**Affiliations:** Department of Electrophysiology, Heart Center Leipzig at University of Leipzig, Leipzig, Germany

**Keywords:** Atrial Fibrillation, Sleep Apnea, Obstructive, Primary Prevention, Aging, Obesity, Arrhythmias, Cardiac

## Abstract

Atrial fibrillation (AF) is a growing health problem worldwide. In recent years, there has been a rising interest in the relationship between sleep disorders and AF. Several studies have reported higher prevalence and incidence rates of AF in patients with obstructive sleep apnea-hypopnea syndrome (OSAHS). However, some believe that OSAHS is not a risk factor for AF; but AF, by itself, is regarded as one of the possible triggers for OSAHS. In this study, the related literature investigating the association between OSAHS and AF was reviewed, and then the possible mechanisms of this interplay were discussed. To conclude, recommendations for further research in this field were presented to researchers and some points were highlighted for physicians.

## Introduction

Atrial fibrillation (AF) is considered as a growing health problem worldwide, affecting approximately 1-4% of the general population [1]. AF is not only associated with increased morbidity and mortality, but also with several disease processes such as stroke and dementia [2-5]. As the incidence rates of AF continues to rise, it is necessary to identify and treat the relevant potentially modifiable risk factors [6]. Hypertension, obesity, smoking, and alcohol consumption have been already approved as possible modifiable risk factors; however, attempts for finding new ones are continuing [7-9]. Recently, there has been a rising interest in the relationship between sleep disorders and AF [10,11]. Both AF and sleep disorders are noticeably increasing in adults, with a considerable prevalence rate in both developing and developed countries [1].Obstructive sleep apnea-hypopnea syndrome (OSAHS), as one of the most prevalent sleep disorders, is defined by recurrent complete or incomplete collapse of the upper airway during sleep, estimated to affect around 5-25% of adult women and men [12,13]. Several studies have even reported a higher prevalence rate of AF in patients with OSAHS; nevertheless, some others have shown that different types of sleep disorders have different impacts on AF risks [14,15]. Moreover, some believe that OSAHS not only does promote AF incidence but also increase the risk of post-ablation AF recurrence [12]. On the other hand, some assume that OSAHS is not a risk factor for AF, but AF by itself should be considered as one of the possible triggers for OSAHS [16]. Several hypotheses have been proposed attempting to clarify the role of the underlying pathophysiological mechanisms of OSAHS on the genesis of AF [17-19]. This paper aimed to review the findings of a variety of studies, especially recently published meta-analyses, and discuss the possible mechanisms regarding the association between OSAHS and AF. Ultimately, recommendations for further research in this field were presented to researchers.

## Does OSAHS Increase AF Risk?

### Observational Studies

The association between OSAHS and AF has been investigated so far in more than ten studies. Based on the present review, most of the published articles have used cohort designs [20-28], followed by cross-sectional [14,15,29,30], case-control [31], and chart-review ones [32]. The first published real-world study investigating sleep disorders associated with AF was conducted by Mooe et al. in 1996 [33]. Accordingly; they showed, for the first time, that sleep-disordered breathing (SDB) with nocturnal hypoxemia might be an independent predictor of AF [33]. During the next years, the researchers even reported that individuals with severe SDB had four-fold odds of AF prevalence (odds ratio [OR]=4.02) [14]. In one other study, a significant relationship was found between the SDB severity and the prevalence of AF (OR: 2.47) among Japanese men [15]. The last cross-sectional study was also conducted in 2016, demonstrating that individuals with moderate-severe SDB had almost two-fold odds of tachyarrhythmia (OR=2.16; P=0.0011) [29]. Regarding the cohort studies (n=9), most of them had shown a significant association between OSAHS and AF incidence (relative risk [RR]: 1.26-2.51). In the very first study, a retrospective cohort of 3542 Olmsted County adults without past or current AF was conducted with a mean follow-up of 4.7 years. They found that OSAHS could predict the AF incidence, mainly in subjects aged <65 years, but not in older age groups [24]. Moreover, in another study, central sleep apnea (CSA) was defined as a predictor of the incidence of AF over a mean of 5.3 years of follow-up, associated with two- to three-fold increased odds of AF incidence [23]. Furthermore, some other studies suggested that different types of OSAHS might have different impacts on AF incidence; even though the effect of some other factors such as old age should not be neglected. In a cohort study of 843 older men, it was reported that CSA (OR: 2.58), but not obstructive apnea or hypoxemia, could predict the incidence of AF [22]. However, some other investigations had suggested that other sleep disorders were also effective concerning the AF incidence. In a multi-ethnic study of atherosclerosis (MESA), among 2048 participants who had undergone polysomnography (PSG), AF was associated with the higher apnea-hypopnea index (AHI, OR: 1.22), and it was more frequent in patients with poor sleep quality as measured by reduced slow wave sleep time [27]. Lastly, in a recently published study, it had been revealed that sleep disruption could steadily predict AF.Moreover, they showed that sleep quality itself might be another significant factor affecting the pathogenesis of AF [34]. Also, for the first time, some evidence of the influence of ethnicity on the relationship between sleep disorders and AF was reported by Ghazi et al. [35] showing that the high risk of OSAHS was related to AF among black but not white individuals. They found that AF prevalence was significantly higher in participants at high risk of OSAHS (9%; n=482/5,359) compared to the low risk of OSAHS (9%; n=1,079/14,992) [35]. Interestingly, some believe that AF might be a possible trigger for sleep apnea syndrome (SAS); however, the evidence is still insufficient. Among the conducted studies in this domain, the prevalence of SAS in groups of patients with AF did not significantly differ from non-AF subjects (32% vs. 29%; P=0.67) [31].

### Meta-Analyses

According to the latest conducted meta-analysis of cohort studies, OSAHS might increase AF risks (RR: 1.70, 95% confidence interval [CI]: 1.53-1.89, P=0.002), and higher severity of central sleep disorders could be related to a higher risk of AF in the general population. Accordingly, this meta-analysis was conducted on a total number of eight studies, involving 603532 individuals with non-sleep disorders and 14799 patients with OSAHS, which seems enough to conclude. However, they failed to evaluate the influence of variables such as the history of cardiovascular disease and body mass index on the risk of AF with a meta-regression analysis, because these variables had always been unavailable in the included studies [13].Another meta-analysis conducted by Youssef et al. had found that the risk of AF was higher in OSAHS group vs. control group (OR; 2.1, 95% CI: 1.84-2.43, P:<0.001); suggesting a higher incidence of AF among patients with definite diagnosis of OSAHS in comparison to no-OSAHS individuals. A total number of nine observational studies were also included in this study with a pooled sample size of 7582 no-OSAHS and 12255 OSAHS patients. However, their meta-analysis had some limitations. First of all, the study design of about 40% of studies was cross-sectional which could limit the ability for concluding the impact of OSAHS on the incidence of AF. They also did not report any quality scores for the included studies, and other confounding factors had not been examined using meta-regression analysis [11].

### Post-Ablation Studies

Catheter ablation has been recognized as an effective treatment method for AF; however, there is much evidence highlighting the role of OSAHS on the risk of post-ablation AF recurrence [36-38]. A prospective study demonstrated a lower post-ablation AF recurrence in those with OSAHS (defining OSAHS as AHI>15/h) compared to individuals without OSAHS (69% vs. 43%; P=0.001) [39]. A meta-analysis by Ng et al. including six observational studies with a pooled sample size of around 4000 patients correspondingly showed that patients without OSAHS (based on PSG reports) had 40% lower risk of post-ablation AF recurrence than those with OSAHS (HR: 1.4; 95% CI: 1.16-1.68) [40]. Also, recently, a meta-analysis conducted by Deng et al. has also revealed that OSAHS could increase the risk of recurrent AF after catheter ablation [12].

## Possible Mechanisms

Different pathophysiologic mechanisms have been taken into consideration for the association between OSAHS and AF, indicating the interplay among multiple factors, rather than a singular one [41]. Changes in blood gases (hypercapnic hypoxia), variations in intrathoracic pressure (negative drop), sympathovagal imbalance (increased ganglionated plexi activity), left atrial dilation, as well as structural and electrical remodeling, and reduced atrial effective refractory period (ERP) are also regarded as the main factors associated with a higher incidence of AF in patients with sleep disorders [42-46].

**Figure 1:**
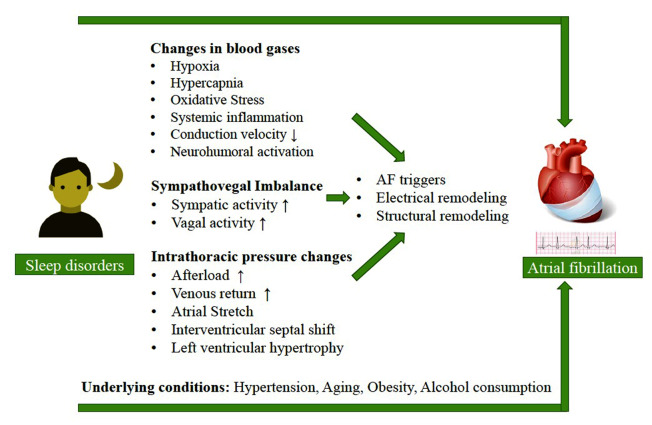
Different possible mechanisms through which sleep disorders may contribute to atrial fibrillation (AF)

Besides, an increase in oxidative stress signaling, as well as inflammatory mediators and neurohumoral activation influenced by the quality of sleep are further deemed as the other possible mediators in the association between OSAHS and AF [47]. As well, some believe that prolonged ERP and slow conduction velocity as the results of hypercapnia, which occurs during OSAHS, can be the other factors affecting the association in the increased risk of AF (Figure 1) [48-52].

Based on the results of human studies; repeated OSAHS over several weeks, can lead to atrial fibrosis, atrial connexin 43 downregulation or lateralization, and even AF-promoting electric changes [42,53]. Remarkably, according to the electroanatomic mapping among patients undertaking AF ablation procedures, it has been demonstrated that increased atrial size and atrial fibrosis, as well as loss of atrial myocardium, or electric uncoupling are the possible AF substrates associated with OSAHS [41,54,55]. However, it seems that the association between OSAHS and AF is much more complicated than it is reported since other factors such as obesity, hypertension, and older age are common in both conditions as triggers [13,18,56,57]. In this respect; obesity is one of the independent predictors of AF which is almost concomitant with OSAHS [58]. Moreover, OSAHS triggers sympathetic nerve activity as well as chemoreflex through repeated episodes of hypoxia, which results in tachycardia and hypertension [59].

## Is OSAHS Treatment Effective for AF?

The amount of evidence about the relationship between the treatment of sleep disorders and AF incidence is limited to observational studies, and no randomized controlled study has been performed so far.

Thus, it is not easy to address these questions: “Does the treatment of sleep disorders have an impact on AF?” and “Does it really decrease the incidence of AF?”. According to the published studies on the effect of the treatment of sleep disorders on AF recurrence after catheter ablation, it seems that continuous positive airway pressure (CPAP) therapies might also be effective for decreasing the burden of AF in patients with sleep disorders [12].

The CPAP as a practical therapy for OSAHS has been insufficiently used, and there have always been uncertainties about the role of CPAP, as a treatment for OSAHS, on the rate of post-ablation AF recurrence [12].

However, beneficial effects of OSAHS treatment with CPAP on AF outcome following ablation have been recently approved in several clinical studies [60,61].

In some investigations, it was suggested that CPAP therapy could result in 70-80% AF free survival in comparison to 36-47% in non-CPAP users following AF ablation [61,62].

Moreover, another study showed that AF recurrence was lower in OSAHS-treated patients compared to the non-treated group (53% vs. 82%; P=0.009) [60].

Deng et al. have also shown in their meta-analysis that CPAP treatment for AF patients with OSAHS might have significantly mitigated AF recurrent risks [12].

Although their pooled sample size was limited to only 1217 participants, they showed that after 16.33±10.34 months of follow-up, 33.5% of the patients had faced recurrent AF, and the recurrence rate was significantly different between the CPAP and non-CPAP groups (24.88% versus 42.47%; RR=0.60; P=0.000).

They also demonstrated that CPAP therapy had decreased the left atrial diameter (LAD) and it had led to an increase in the left ventricular ejection fraction (LVEF) [12].

Besides, it was argued that more clinical randomized controlled trials were required to further support and confirm their findings, mainly due to the small sample size of included studies (3 out of 10 articles).

## Future Directions

More information is needed concerning accuracy as well as impact, and above all cost-effectiveness of the implementation of OSAHS testing and its treatment strategies in daily AF care.

Likewise, more randomized controlled studies are required to confirm the success rate of OSAHS treatment on different parameters in AF burden and AF ablation outcomes.

Moreover, the effects of alternative therapeutic options for sleep disorders such as weight reduction or position modification devices in comparison to CPAP on AF burden and symptoms seem to be of great significance.

Furthermore, more studies are required to indicate which AHI thresholds should be considered as indications for OSAHS treatment in AF patients. Finally, there is still an important question to be answered; i.e., how ethnicity, regional differences, gender, as well as age may affect the association between AF and sleep disorders.

## Conclusion

It seems evident that the prevalence and incidence of AF are higher in individuals with OSAHS, especially in middle-aged adults and those with other risk factors. However, the efficacy of any therapeutic interventions among patients with OSAHS in terms of decreasing the risk of AF is not clear yet. Therefore, advising patients receiving OSAHS therapeutic interventions only for reducing the risk of AF needs additional evidence. Moreover, while OSAHS patients are being encountered with greater risks of AF recurrence after catheter ablation than those without OSAHS, the treatment of OSAHS using CPAP is recommended.

## Conflict of Interest

G.H. and N.D. report research grants from Abbott, Biotronik, Boston Scientific and Medtronic to the institution without personal financial benefits. AA. and A.S.SH have no conflict of interest.
